# Utility of Different Surrogate Enzyme-Linked Immunosorbent Assays (sELISAs) for Detection of SARS-CoV-2 Neutralizing Antibodies

**DOI:** 10.3390/jcm10102128

**Published:** 2021-05-14

**Authors:** Niko Kohmer, Cornelia Rühl, Sandra Ciesek, Holger F. Rabenau

**Affiliations:** 1 Institute for Medical Virology, University Hospital, Goethe University Frankfurt am Main, 60596 Frankfurt, Germany; Niko.Kohmer@kgu.de (N.K.); Conny.Ruehl@kgu.de (C.R.); Sandra.Ciesek@kgu.de (S.C.); 2 German Centre for Infection Research, External Partner Site, 60323 Frankfurt, Germany; 3 Fraunhofer Institute for Molecular Biology and Applied Ecology (IME), Branch Translational Medicine and Pharmacology, 60596 Frankfurt, Germany

**Keywords:** SARS-CoV-2, surrogate ELISA, PRNT, neutralizing antibodies

## Abstract

The plaque reduction neutralization test (PRNT) is a preferred method for the detection of functional, SARS-CoV-2 specific neutralizing antibodies from serum samples. Alternatively, surrogate enzyme-linked immunosorbent assays (ELISAs) using ACE2 as the target structure for the detection of neutralization-competent antibodies have been developed. They are capable of high throughput, have a short turnaround time, and can be performed under standard laboratory safety conditions. However, there are very limited data on their clinical performance and how they compare to the PRNT. We evaluated three surrogate immunoassays (GenScript SARS-CoV-2 Surrogate Virus Neutralization Test Kit (GenScript Biotech, Piscataway Township, NJ, USA), the TECO^®^ SARS-CoV-2 Neutralization Antibody Assay (TECOmedical AG, Sissach, Switzerland), and the Leinco COVID-19 ImmunoRank™ Neutralization MICRO-ELISA (Leinco Technologies, Fenton, MO, USA)) and one automated quantitative SARS-CoV-2 Spike protein-based IgG antibody assay (Abbott GmbH, Wiesbaden, Germany) by testing 78 clinical samples, including several follow-up samples of six BNT162b2 (BioNTech/Pfizer, Mainz, Germany/New York, NY, USA) vaccinated individuals. Using the PRNT as a reference method, the overall sensitivity of the examined assays ranged from 93.8 to 100% and specificity ranged from 73.9 to 91.3%. Weighted kappa demonstrated a substantial to almost perfect agreement. The findings of our study allow these assays to be considered when a PRNT is not available. However, the latter still should be the preferred choice. For optimal clinical performance, the cut-off value of the TECO assay should be individually adapted.

## 1. Introduction

The severe acute respiratory syndrome coronavirus 2 (SARS-CoV-2) is the causative agent of the ongoing coronavirus disease 2019 (COVID-19), which was first reported in Wuhan, China in late 2019 [1,2]. As of mid-March 2021, confirmed SARS-CoV-2 infections exceeded 119 million worldwide and cases continue to rise [3]. Despite the early detection of new infections and rigorous infection control measures, the presence or absence of protective immunity will affect future spread, illness severity, and public health response [4]. In a large study conducted in Israel, the recently available BNT162b2 mRNA Covid-19 Vaccine (BioNTech/Pfizer) reduced the risk of symptomatic COVID-19 disease by 94% and the risk of asymptomatic SARS-CoV-2 infection by 90% [5]. This suggests that vaccinated individuals are less likely to transmit the pathogen. However, to date, there are limited data on the duration and nature of immunity generated in response to SARS-CoV-2 infection or after vaccination. To measure the humoral mediated immune response caused by SARS-CoV-2, several commercially available and laboratory-developed antibody tests have been established [6,7,8]. The sensitivity and specificity of these assays vary widely, depending on the used technology, detected antibody class, disease severity, and moment of testing in the infection phase.

The plaque reduction neutralization test (PRNT) is one of the preferred methods for the detection of functional, coronavirus-specific neutralizing antibodies from serum samples [9]. Neutralizing antibodies most commonly target the receptor-binding domain (RBD) region of the SARS-CoV-2 spike (S) protein, block viral entry, and are therefore of particular interest in determining whether antibodies offer protective immunity [10]. However, the PRNT is a biological assay that is hands-on- and time-intensive and can only be performed for smaller sample sizes by experienced personnel in a BSL-3 laboratory. Sample throughput may be increased by using microneutralization assays. Pseudovirus-based neutralization assays use replication deficient virus and do not require former safety measures [11,12,13]. They are also widely used and accepted, but they are also labor-intensive and not suitable for the analysis of a large cohort of subjects. Alternatively, surrogate enzyme-linked immunosorbent assays (sELISAs) have been developed or are under development [14]. These assays work according to the principle of competitive binding: anti-SARS-CoV-2 neutralizing antibodies block an enzyme-labeled S-RBD protein from binding its natural ligand, the angiotensin-converting enzyme 2 (ACE2), pre-coated on a microtiter plate. Surrogate ELISAs are high-throughput capable and can be performed under standard laboratory safety conditions [15,16]. However, there are limited data on their performance when testing specimens of individuals with past SARS-CoV-2 infection or follow-up samples of SARS-CoV-2 vaccinated individuals and especially how they compare to the PRNT. 

The aim of our study was to evaluate the clinical performance of three manual surrogate ELISAs and one automated quantitative SARS-CoV-2 S protein-based IgG antibody assay by comparing test results to the PRNT conducted in parallel. We analyzed serum follow-up samples taken at different timepoints of individuals vaccinated with two doses of BNT162b2 (BioNTech/Pfizer) and a selection of clinical specimens, including follow-up serum samples of individuals with past SARS-CoV-2 infection, external quality assessment (EQA) samples, and in the PRNT SARS-CoV-2 seronegative tested samples.

## 2. Materials and Methods

### 2.1. Serum Samples

We collected follow-up serum samples (*n* = 29) at different timepoints from 6 individuals vaccinated with two doses of BNT162b2 (BioNTech/Pfizer). Additional analyzed specimens (*n* = 50) were a selection of follow-up samples of SARS-CoV-2 infected individuals (serologically and/or PCR confirmed), including samples of individuals infected with SARS-CoV-2 carrying the N501Y and del69/70 (*n* = 6, including two confirmed B.1.1.7 Variants of Concern (VOC)) and del69/70 spike key mutations (*n* = 1). Furthermore, we tested a sample containing Bamlanivimab (*n* = 1), a man-made monoclonal neutralizing antibody similar to the antibodies of patients who recovered from COVID-19 [17]. In addition, external quality assessment (EQA) samples (*n* = 4) and routinely in the PRNT negative tested samples were tested. The SARS-CoV-2 infected individuals had an asymptomatic to severe clinical course; some required a hospital stay at the intensive care unit. 

### 2.2. Plaque Reduction Neutralization Test (PRNT)

To test for the neutralizing capacity of SARS-CoV-2 specific antibodies, Caco-2 cells (human colon carcinoma cells, ATCC DSMZ ACC-169 (American Type Culture Collection, Manassas, VA, USA)) were seeded on a 96-well plate 3 to 5 days prior to infection. Two-fold dilutions of the test sera beginning with a 1:10 dilution (1:10; 1:20; 1:40; 1:80; 1:160; 1:320; 1:640; and 1:1280) were made in culture medium (minimum essential medium, MEM; Sigma-Aldrich, St. Louis, MO, USA) before being mixed 1:1 with 100 TCID_50_ (Tissue culture infectious dosis 50) of reference virus (SARS-CoV-2 FFM1 isolate). FFM1 was isolated from a patient at University Hospital Frankfurt who was tested positive for SARS-CoV-2 by PCR. Virus-serum mixture was incubated for one hour at 37 °C and transferred onto the cell monolayer. Virus-related cytopathic effects (CPE) were determined microscopically 48 to 72 hours post infection. To determine a potential neutralizing ability of patient serum, CPE at a sample dilution of 1:10 is defined as a “borderline” result, while a CPE at a dilution of ≥ 1:20 is defined as a positive result.

### 2.3. Immunoassay System

The Abbott SARS-CoV-2 IgG II Quant, a chemiluminescent microparticle based immunoassay (CMIA), was used on the Abbott Alinity i platform (Abbott GmbH, Wiesbaden, Germany), according to the manufacturers’ recommendation. This assay measures antibody targeted against the SARS-CoV-2 spike (S protein) receptor-binding domain (RBD). Test results are expressed as standardized binding antibody units (BAU)/mL, calibrated to the WHO International Standard for anti-SARS-CoV-2 immunoglobulin (human) (NIBSC Code 20-136) [18]. The manufacturer’s cut-off for positivity is set to 7.1 BAU/mL. We defined the range from 5.68 to 8.52 BAU/mL as “borderline” and >8.52 BAU/mL as cut-off for positivity.

### 2.4. Surrogate ELISAs

We used the ELISA-based GenScript SARS-CoV-2 Surrogate Virus Neutralization Test Kit (GenScript Biotech, Piscataway Township, USA), the TECO^®^ SARS-CoV-2 Neutralization Antibody Assay (TECOmedical AG, Sissach, Switzerland), and the Leinco COVID-19 ImmunoRank™ Neutralization MICRO-ELISA (Leinco Technologies, Fenton, MO, USA) in our study. All assays were used in an identical manner, according to the manufacturers’ recommendation. Samples were diluted in sample buffer and incubated at 37° for 30 min in the provided 96-well microtiter plates followed by each protocols’ washing and incubation cycles, including controls and required reagents. The microtiter plates are coated with the “host cell receptor” angiotensin-converting enzyme 2 (ACE2). Samples containing SARS-CoV-2 neutralizing antibodies block the protein–protein reaction between ACE2 and the added (S)-RBD–horseradish peroxidase conjugate. The reduced change of color upon the addition of chromogenic substrate can be measured photometrically. Optical density (OD) was measured for all assays at 450 nm using the microplate reader of a VIRCLIA^®^ automation system. The signal-to-cut-off ratio was calculated and values expressed and interpreted according to each manufacturer’s protocol. For the TECO^®^ SARS-CoV-2 Neutralization Antibody Assay and after consultation with the manufacturer, the cut-off to positivity was adapted to ≥30% inhibition.

### 2.5. Statistical Analysis

The agreement between the examined SARS-CoV-2 antibody assays and the PRNT results was evaluated using Cohen’s weighted kappa index (K value) [19]. K value interpretations were categorized as follows: <0.20 is poor, 0.21–0.40 is fair, 0.41–0.60 is moderate agreement, 0.61–0.80 is substantial agreement, and 0.81–1.00 is almost perfect agreement [20]. Clopper–Pearson confidence intervals for sensitivity/specificity were calculated using MedCalc (MedCalc Software, Ostend, Belgium).

## 3. Results

**Figure 1:**
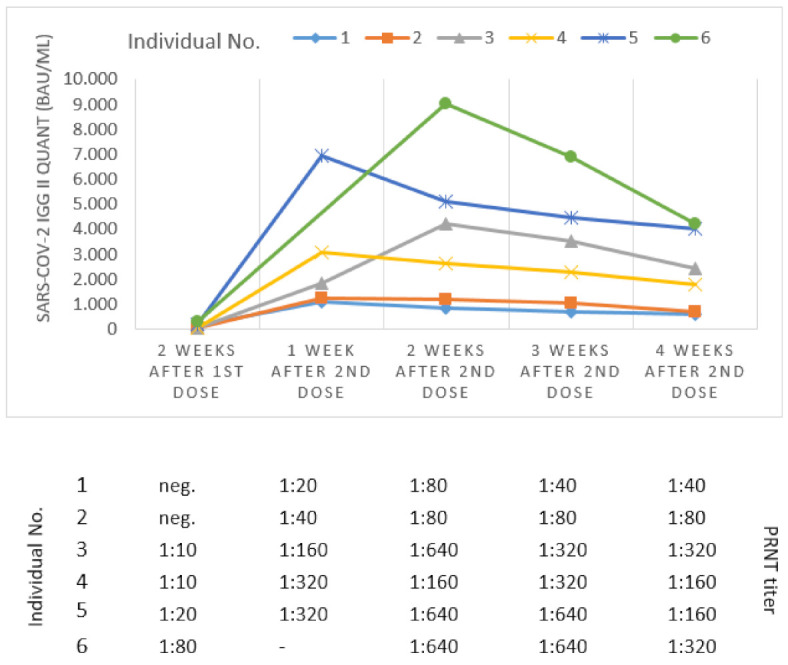
SARS-CoV-2 (RBD) IgG and PRNT antibody titers in follow-up serum samples of six individuals vaccinated with BNT162b2. neg. = negative; - not tested.

Between December 2020 and February 2021, we collected follow-up serum samples of six individuals, which were vaccinated with BNT162b2 (BioNTech/Pfizer) according to the manufacturer’s recommendation. To measure the humoral mediated immune response, samples were tested for SARS-CoV-2 specific antibodies using the SARS-CoV-2 IgG II Quant (Abbott) assay. Results are shown in Figure 1.

Two weeks after the first dose, rather low SARS-CoV-2 IgG antibody titers (<2300 BAU/mL) could be detected in all individuals. One week after the second dose, a 7.6 to 34.1-fold increase in titers could be observed, reaching a maximum of 1161.5 to 6949.2 BAU/mL. The first follow-up serum sample for Individual No. 6, taken two weeks after the second dose, reached a maximum of 9006 BAU/mL, which was the highest titer observed in our cohort. From two to four weeks after the second dose, the antibody titers of all examined individuals waned in an identical manner. After the second dose and for individuals No. 1 and 2 (>50 years old) with no reported side effects for both doses, low antibody titers were observed. For individuals No. 3 to 6 (<50 years old), much higher antibody titers could be observed in follow-up serum samples taken after the second dose. Individuals No. 3, 4, and 6 reported side effects such as fever, red and swollen injection site, and locally swollen lymph nodes after the first and/or second dose. Generated titers are in line with the results of the PRNT conducted in parallel (Table S1). PRNT results ranged from no detectable antibodies for the first follow-up samples to a peak of maximum 1:640 for the next follow-up samples. A range between 1:40 and 1:320 could be observed for the last follow-up samples. 

In addition, a selection of specimens (*n* = 78), including the follow-up samples of the vaccinated individuals was analyzed using three manual surrogate ELISAs and the SARS-CoV-2 IgG II Quant (Abbott) and compared to the PRNT, which was conducted in parallel. Overall sensitivity ranged between 93.8 and 100% and specificity ranged between 73.9 and 91.3% (Table 1).

The GenScript, TECO, and Abbott assays demonstrated the highest sensitivity of 100%, which was followed by the Leinco test with a sensitivity of 93.8%. The three samples not detected as positive in the Leinco assay were only weakly positive in the PRNT with a titer of 1:20, including two samples of individuals with past SARS-CoV-2 infection (samples No. 34 and 71) and one EQA sample of a vaccinee (sample No. 55). When testing only the high level (≥1:80) PRNT positive samples, the Leinco test also showed a sensitivity of 100%. The highest specificity of 91.3% demonstrated the Leinco test, followed by the GenScript and TECO tests with a specificity of 87% and 78.3%. The Abbott test showed a specificity of 73.9%, which was the lowest specificity observed in our study. As recommended by the manufacturer, we set the inhibition cut-off value of the TECO test to ≥30% inhibition for a better sensitivity to specificity ratio, in our study in favor for specificity. For the PRNT “borderline” (1:10) tested samples, the Abbott, GenScript, and TECO tests tended to generate positive test results, while the Leinco test tended to generate negative test results.

**Figure 2:**
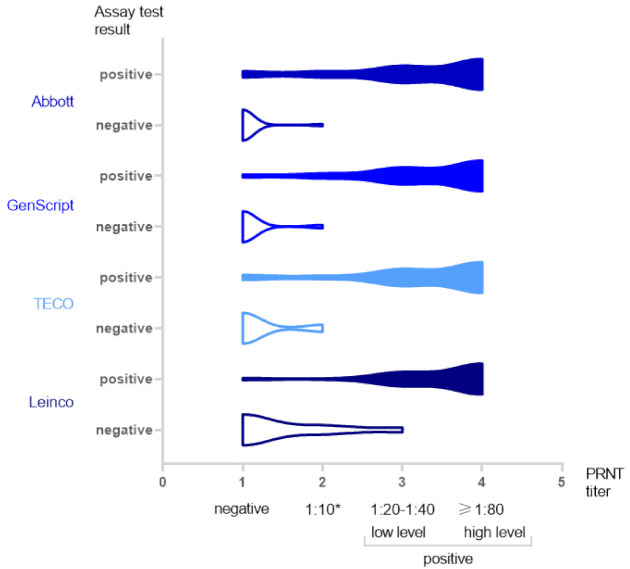
Distribution of test results of the examined SARS-CoV-2 antibody assays in relation to the PRNT results. The width of each violin reflects the number of generated results: wide = high number of results, narrow = low number of results; * “borderline” result.

The distribution of the test results of the examined SARS-CoV-2 antibody assays in relation to the PRNT results is shown in Figure 2. When compared to the PRNT, the examined surrogate ELISAs detected samples as positive in a similar fashion, the number of positive results increases from PRNT negative toward the high level (≥1:80) of tested samples. The Leinco assay tested a decreasing number of samples as negative, ranging from PRNT negative to low level positive samples. The Abbott, GenScript, and TECO assays tested samples as negative ranging only from PRNT negative to “borderline” tested samples. 

**Figure 3:**
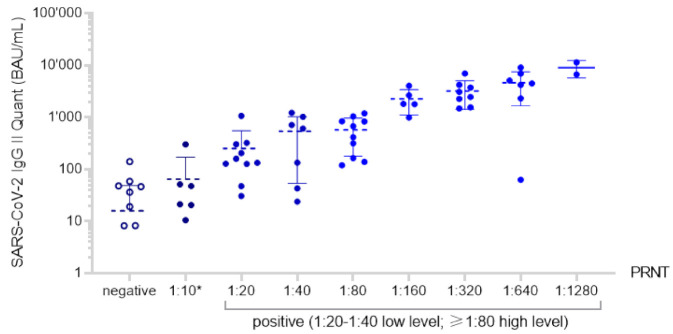
Distribution of SARS-CoV-2 IgG II Quant test results (BAU/mL) in relation to the results of the PRNT conducted in parallel, including mean and standard deviation bars. PRNT test results ≥1:10 are shown as filled data point symbols and negative results are shown as empty data point symbols. Fifteen samples were negative tested in the SARS-CoV-2 IgG II Quant as they did not generate a signal. As a result of the logarithmic scale of the y-axis, they are not depicted in the figure. * “borderline” result.

The distribution of titers (BAU/mL) generated by the SARS-CoV-2 IgG II Quant (Abbott) in relation to the PRNT results is shown in Figure 3. In accordance with the manufacturer’s aim to standardize measured values by calibrating the assay to a gold standard method, we could observe a linear trend: the results show a good correlation to the PRNT titers, with a particularly high precision for the ≥1:160 tested PRNT samples.

**Figure 4:**
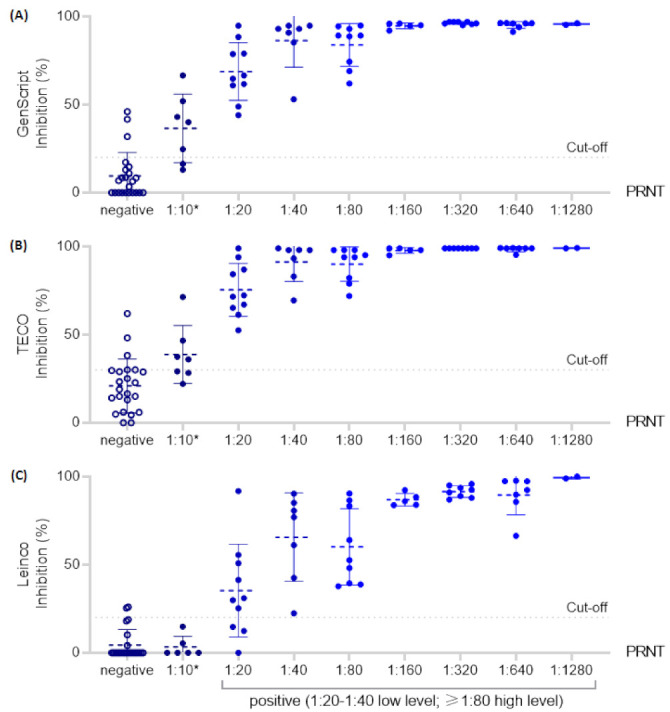
Distribution of the inhibition (%) of each examined sELISA in relation to the PRNT conducted in parallel, including mean and standard deviation bars. PRNT test results ≥1:10 (“borderline” or positive results) are shown as filled data point symbols and negative results are shown as empty data point symbols. Error bars outside the axis limits are not shown. (**A**) = GenScript; (**B**) = TECO; (**C**) = Leinco assay; * “borderline” result.

The distribution of the inhibition (%) examined in the surrogate ELISAs in relation to the results of the PRNT is shown in Figure 4. The GenScript (A), TECO (B) and Leinco (C) assays demonstrated comparable results. A particularly high precision could be observed in PRNT samples with a titer ≥1:160.

Cohen’s weighted kappa coefficient between the SARS-CoV-2 antibody assays and PRNT results showed an almost perfect agreement with a kappa for the GenScript test of 0.9 > Leinco test of 0.841 > TECO test of 0.830 and a substantial agreement with a kappa for the Abbott test of 0.793 (Table 2).

The sample containing the monoclonal antibody Bamlanivimab was tested in the PRNT as high level positive with a titer >1:1280, in the quantitative Abbott assay >11,360 BAU/mL, and in the three surrogate ELISAs as clearly positive. Two in the PRNT positive tested samples (samples No. 11 and 12) from individuals infected with SARS-CoV-2 B.1.1.7 VOC and one sample (sample No. 13) of an individual infected with SARS-CoV-2 harbouring the del69/70 spike mutation were detected as positive in all four examined assays.

## 4. Discussion

In our study, we investigated the value of different SARS-CoV-2 surrogate enzyme-linked immunosorbent assays (sELISAs) for the detection of neutralization-competent antibodies compared to a cell culture-based method (PRNT) and a quantitative anti-RBD spike IgG assay. The PRNT is one of the preferred methods to test anti-SARS-CoV-2 neutralization activity, but it is no longer regarded as the only “gold standard” [21,22]. In fact, also microneutralization assays and pseudovirus tests are widely accepted [23,24,25]. 

Of the surrogate ELISAs examined in our study, the GenScript assay demonstrated the highest sensitivity of 100% and a specificity of 87%, which is comparable to data from other studies. In the literature, also, testing clinical specimens and when the PRNT is used as the reference method, sensitivity ranges from 77 to 100% and specificity ranges from 69.5 to 100% [15,26,27,28]. When serum follow-up samples taken >14 days after rRT-PCR positivity were analyzed, the GenScript assay demonstrated a sensitivity of 97.3% and a specificity of 99.4% [16]. In a Belgian cohort, when the Luminex multiplex immunoassays was used as the reference method, the sensitivity was 94% [29]. There are no articles regarding the clinical performance of the Leinco and TECO assays. The manufacturer of the TECO assay states a sensitivity of 99.03% and a specificity of 100% when compared to the PRNT. After consultation with the manufacturer, we adapted the cut-off to positivity to ≥30% inhibition. Without adaptation, this assay would have demonstrated a rather low specificity in our investigation. When setting the cut-off value to ≥31% or ≥49% inhibition in our study, the specificity could be further increased to 87% or even 95.7% with no change in sensitivity. The cut-off values for the GenScript and Leinco assays were also used according to the manufacturers’ recommendation, but they may also be adapted individually, which might result in a different sensitivity and specificity. The accuracy of the Leinco assay according to the package insert, when a standard live virus focus reduction neutralization test (FRNT) was used as reference, was given with a positive and negative percent agreement of 92% and 100%. In our study there was no difference between PRNT positive tested samples and the positive test results of the examined assays for samples from vaccinees and clinical samples, with the exception of three samples (two samples of individuals with past SARS-CoV-2 infection and one vaccinee) tested negative in the Leinco assay. Therefore the groups were not analyzed separately. In addition, the panel of PRNT negative tested samples was mostly the same.

The standardized SARS-CoV-2 IgG II Quant (Abbott) demonstrated a good correlation with the PRNT in our study. The manufacturer also states a 100% percent positive agreement with the PRNT by testing 86 samples in its “Reagent Instructions for Use”. If the cut-off value is set to match high level positive PRNT samples, where the assay demonstrated a particularly high precision in our study, it may be used as a standalone assay in determining SARS-CoV-2 humoral mediated immunity where a PRNT is not available.

Our observation of high SARS-CoV-2 specific antibody titers in individuals reporting side effects after receiving the COVID-19 vaccine is in concordance with a positive correlation between disease severity after SARS-CoV-2 infection and level of detected antibodies [30,31,32]. However, overall negative economic, individual, and health system consequences after SARS-CoV-2 infection outweigh reported side effects after vaccination by far. Studies also show that neutralizing antibody levels decline after the acute phase of COVID-19 or plateau and remain detectable for several months [33]. Similar kinetics were also observed in a study by Grupel et al. where IgG antibody levels in BNT162b2 vaccinated healthcare workers were examined [34]. Local reactogenicity was also higher in the safety and efficacy trail of BNT162b2 in individuals younger than 55 years of age [35]. The correlation between younger age (<50 years) of vaccinated individuals in our study and high antibody titers may be due to a decline in immune response (immunosenescence) with increasing age [36]. An age-dependent immune response to the BNT162b2 vaccination could also be observed in the study by Grupel et al. and a study by Müller et al. [34,37]. Grupel et al. could observe a statistically significant difference in IgG levels between vaccinees over and younger 50 years of age. Although it is not entirely clear how long SARS-CoV-2 humoral mediated immunity lasts or how long specific antibodies are detectable, antibody testing can help to easily and cost-effectively determine the current immune status of an individual. 

The observation that the sample containing the monoclonal antibody Bamlanivimab, targeting the RBD spike protein of SARS-CoV-2 [17], tested positive at a high level in the PRNT and clearly positive in the quantitative Abbott assay and the three surrogate ELISAs, highlights the high specificity of this antibody preparation and the potential capability of these tests in the detection of neutralization-competent antibodies. In our study, two in the PRNT-positive tested samples from individuals infected with SARS-CoV-2 B.1.1.7 VOC and the sample of one individual infected with SARS-CoV-2 harbouring the del69/70 spike mutation were detected as positive in all examined assays. However, as emerging mutations are in the RBD region of the SARS-CoV-2 S protein, which is the target of many SARS-CoV-2 specific antibody assays, their sensitivity should continuously be assessed.

In addition to a humoral mediated immune response, T cell-mediated immune response plays a role in SARS-CoV-2 infection and the specific cellular immunity may be more stable and longer lasting than humoral immunity [38,39]. This is particularly of interest regarding the immunity of recovered patients with low or no detectable neutralizing antibodies [40]. However, the mechanism of immune memory is complex, and the source of SARS-CoV-2 long-term protective immunity not defined in humans [41]. 

Our study has clear limitations, including a limited sample size. Therefore, the results generated in this study may be used for an orientation. The major strength of this work is the testing of clinical specimens and the detailed analysis of follow-up samples of vaccinated individuals by three recently available surrogate ELISAs, the standardized quantitative SARS-CoV-2 IgG antibody assay, and the comparison to the PRNT as a reference method. Correlation between test results of the broadly available and simple to perform SARS-CoV-2 antibody assays, surrogate ELISAs, and the PRNT is an important indication of their clinical and public health utility in determining humoral mediated immunity. However, more samples need to be tested to get a clearer picture, especially to reveal a potential difference between the detection of samples from individuals with past SARS-CoV-2 infection and vaccinees.

The high sensitivity, moderate specificity, and substantial to almost perfect agreement of the examined surrogate SARS-CoV-2 antibody assays when compared to the PRNT as reference method allow these assays to be used as alternative testing methods in determining humoral mediated immunity to SARS-CoV-2 where a PRNT is not available. A huge advantage over the classical PRNT is the turn-around time of only 3–5 h versus 3–5 days and no need of a BSL-3 laboratory. However, for the TECO assay, the cut-off value had to be adapted individually. The quantitative Abbott assay, although no neutralization surrogate test, demonstrated a good correlation with the PRNT and would be a good option if automation is desired.

## Figures and Tables

**Table 1:** Overall sensitivity and specificity of the examined SARS-CoV-2 antibody assays in comparison to the PRNT.

	PRNT		GenScript SARS-CoV-2 Surrogate Virus Neutralization Test Kit	TECO^®^ SARS-CoV-2 Neutralization Antibody Assay	Leinco COVID-19 ImmunoRank™ Neutralization MICRO-ELISA	SARS-CoV-2 IgG II Quant (Abbott)
Sensitivity (%)	all positive tested samples (titer ≥ 1:20)	*n* = 48	100% (48/48) (92.6–100% [95% CI])	100% (48/48) (92.6–100% [95% CI])	93.8 (45/48) (82.8–98.7% [95% CI])	100% (48/48) (92.6–100% [95% CI])
	low level (titer 1:20–1:40)	*n* = 17	100% (17/17) (80.5–100% [95% CI])	100% (17/17) (80.5–100% [95% CI])	82.4% (14/17) (56.6–96.2% [95% CI])	100% (17/17) (80.5–100% [95% CI])
	high level (titer ≥ 1:80)	*n* = 31	100% (31/31) (88.8–100% [95% CI])	100% (31/31) (88.8–100% [95% CI])	100% (31/31) (88.8–100% [95% CI])	100% (31/31) (88.8–100% [95% CI])
	“borderline” (titer 1:10)	*n* = 7	5 × positive/ 2 × negative	4 × positive/ 3 × negative	1 × positive/ 6 × negative	6 × positive/ 1 × negative
Specificity (%)	PRNT negative samples	*n* = 23	87% (20/23) (66.4–97.2% [95% CI])	78.3% (18/23) (56.3–92.5% [95% CI])	91.3% (21/23) (72–98.9% [95% CI])	73.9% (17 */23) (51.6–89.8% [95% CI])

* two equivocal results were considered as negative.

**Table 2:** Cohen’s weighted kappa coefficient between the examined SARS-CoV-2 antibody assays and PRNT results.

	GenScript SARS-CoV-2 Surrogate Virus Neutralization Test Kit	TECO^®^ SARS-CoV-2 Neutralization Antibody Assay	Leinco COVID-19 ImmunoRank™ Neutralization MICRO-ELISA	SARS-CoV-2 IgG II Quant (Abbott)
weighted kappa	0.9	0.830	0.841	0.793
standard error	0.056	0.072	0.068	0.079
95% CI	0.79–1	0.688–0.971	0.707–0.975	0.638–0.948

Kappa < 0.20: poor agreement; 0.21–0.40: fair agreement; 0.41–0.60: moderate agreement; 0.61–0.80: substantial agreement; 0.81–1.00: almost perfect agreement.

## Data Availability

The data presented in this study are available in the Supplementary Material.

## Supplementary Materials

The following are available online at https://www.mdpi.com/article/10.3390/jcm10102128/s1, Table S1: Examined clinical specimens and generated results in the surrogate ELISAs, the quantitative IgG antibody assay and PRNT.
