# Supplementary material for: Utility of Different Surrogate Enzyme-Linked Immunosorbent Assays (sELISAs) for Detection of SARS-CoV-2 Neutralizing Antibodies
Source: J Clin Med. 2021 May 14;10(10):2128. doi: 10.3390/jcm10102128 (PMC8157164; doi:10.3390/jcm10102128)
Supplement: Supplementary file 1 [file jcm-10-02128-s001.zip › jcm-1176849-supplementary.pdf]

Table 1. Examined clinical specimens and generated results in the surrogate ELISAs, the quantitative IgG antibody assay and PRNT.

No.	PRNT (titer 1:x)	GenScript SARS-CoV-2 Surrogate Virus Neutralization Test Kit Inhibition (%)	Leinco COVID-19 ImmunoRank™ Neutralization MICRO-ELISA Inhibition (%)	TECO® SARS-CoV-2 Neutralization Antibody Assay Inhibition (%)	SARS-CoV-2 IgG II Quant (Abbott) BAU/mL	Details
1	neg.	neg.	neg.	70	8.17	
2	10	43	14.9	77	47.60	Individual No. 4, 3 weeks after first dose (BNT162b2)
3	neg.	17.2	neg.	78	140.18	Individual No.1, 3 weeks after first dose (BNT162b2)
4	20	64.7	41.4	88	203.77	Individual No. 5, 3 weeks after first dose (BNT162b2)
5	80	88.7	64	95	315.10	Individual No. 6, 3 weeks after first dose (BNT162b2)
6	neg.	31.9	18	78	47.85	Individual No.2, 3 weeks after first dose (BNT162b2)
7	neg.	41.7	25.4	78	58.36	
8	neg.	neg.	neg.	73	36.25	Individual No. 1, 1 week after first dose (BNT162b2)
9	10	16.4	neg.	73	21.16	Individual No.3, 1 week after first dose (BNT162b2)
10	640	91.3	66.4	96	62.48	
11	40	85.3	61.1	93	23.77	infection with SARS-CoV-2 including N501Y and del69/70 Spike mutations
12	20	66.5	25.3	83	133.05	infection with SARS-CoV-2 including N501Y and del69/70 Spike mutations
13	20	61.7	29.9	83	127.80	infection with SARS-CoV-2 including del69/70 Spike mutation
14	320	96	95	99	3083.67	Individual No.4, 4 weeks after first dose (BNT162b2)
15	20	94.8	91.8	98	1061.45	Individual No. 1, 1 week after second dose (BNT162b2)
16	320	96.1	93.6	99	6949.20	Individual No. 5, 1 week after second dose (BNT162b2)
17	40	94.8	85.1	98	1019.28	
18	640	96.3	97.7	99	2302.81	
19	neg.	neg.	neg.	57	neg.	
20	neg.	neg.	neg.	61	neg.	
21	neg.	neg.	neg.	63	neg.	
22	neg.	neg.	neg.	48	neg.	
23	neg.	neg.	neg.	60	neg.	
24	neg.	neg.	neg.	57	neg.	
25	neg.	neg.	neg.	44	neg.	
26	20	78.7	55.6	83	126.99	
27	10	24.7	neg.	40	neg.	
28	10	52	neg.	55	51.39	
29	160	92	83.8	94	986.62	
30	1280	95.2	98.9	99	6672.01	
31	80	89.2	48.1	77	412.21	
32	20	88.4	50.8	88	302.18	
33	neg.	46	26	45	46.29	
34	20	60.9	14.7	51	159.47	
35	40	90.8	42.6	65	134.33	

36	640	96.3	97.3	99	9006.07	Individual No.6, 2 weeks after second dose (BNT162b2)
37	40	94.8	90.3	98	1217.79	Individual No.2, 1 week after second dose (BNT162b2)
38	160	94.6	88.4	97	1811.78	Individual No.3, 1 week after second dose (BNT162b2)
39	80	74.3	39.4	70	138.17	
40	640	96.2	97.4	99	4198.66	Individual No.3, 2 weeks after second dose (BNT162b2)
41	80	94.9	90.4	99	1186.13	Individual No.2, 2 weeks after second dose (BNT162b2)
42	neg.	3.5	neg.	14	neg.	
43	neg.	14.7	neg.	26	neg.	
44	10	66.5	40	68	300.76	
45	neg.	6.5	neg.	11	neg.	
46	neg.	6.9	neg.	16	neg.	
47	neg.	8.4	neg.	13	neg.	
48	>1280	96.2	>100	99	neg.	Sample including Bamlanivimab
49	neg.	8.5	neg.	9.6	neg.	
50	160	95.9	92.3	99	2643.05	Individual No. 4, 2 weeks after second dose (BNT162b2)
51	640	96.1	89.8	99	5104.90	Individual No. 5, 2 weeks after second dose (BNT162b2)
52	80	94.4	86.5	96	832.83	Individual No.1, 2 weeks after second dose (BNT162b2)
53	320	95.7	95.8	99	3725.94	
54	80	89.2	52.6	84	830.98	EQA sample 1
55	20	48.9	neg.	40	30.53	EQA sample 2
56	neg.	8.6	neg.	15	neg.	EQA sample 3
57	20	79	31	69	320.78	EQA sample 4
58	80	93	83.3	96	1040.15	Individual No.2, 3 weeks after second dose (BNT162b2)
59	640	94	85.7	99	4465.05	Individual No.5, 3 weeks after second dose (BNT162b2)
60	320	95	87.9	99	2258.79	Individual No.4, 3 weeks after second dose (BNT162b2)
61	640	96	92.4	99	6873.23	Individual No. 6, 3 weeks after second dose (BNT162b2)
62	40	93	80.7	95	711.99	Individual No.1, 3 weeks after second dose (BNT162b2)
63	320	97	92.6	99	3541.04	Individual No.3, 3 weeks after second dose (BNT162b2)
64	10	40	neg.	23	20.43	infection with SARS-CoV-2 including N501Y and del69/70 Spike mutations
65	neg.	13	18.9	18	19.14	infection with SARS-CoV-2 including N501Y and del69/70 Spike mutations
66	neg.	neg.	4.2	15	neg.	infection with SARS-CoV-2 including N501Y and del69/70 Spike mutations
67	neg.	11	10.2	12	neg.	infection with SARS-CoV-2 including N501Y and del69/70 Spike mutations
68	40	53	22.4	33	42.74	
69	10	13	5.4	15	10.51	
70	80	62	38.8	52	119.00	
71	20	44	12.4	34	47.29	
72	80	69	37.8	42	164.01	
73	320	97	87	99	1550.78	

74	160	95	86	98	1779.12	Individual No. 4, 4 weeks after second dose (BNT162b2)
75	160	96	84	98	4014.91	Individual No. 5, 4 weeks after second dose (BNT162b2)
76	40	93	77	94	607.90	Individual No. 1, 4 weeks after second dose (BNT162b2)
77	320	97	89	99	4194.68	Individual No. 6, 4 weeks after second dose (BNT162b2)
78	320	97	91	99	2441.97	Individual No. 3, 4 weeks after second dose (BNT162b2)
79	80	-	-	-	671.04	Individual No. 2, 4 weeks after second dose (BNT162b2)

Interpretation PRNT: 1:10 = "borderline", ≥1:20 = positive; GenScript and Leinco assays: ≥20 = positive, <20 = negative; TECO assay: ≥77 = positive, <77 = negative; Abbott assay: <5.68 = negative, 5.68-8.52 = "borderline", >8.52 = positive; neg. = negative; - not tested
